# Differentiated HIV care in South Africa: the effect of fast‐track treatment initiation counselling on ART initiation and viral suppression as partial results of an impact evaluation on the impact of a package of services to improve HIV treatment adherence

**DOI:** 10.1002/jia2.25409

**Published:** 2019-11-05

**Authors:** Sophie JS Pascoe, Matthew P Fox, Amy N Huber, Joshua Murphy, Mokgadi Phokojoe, Marelize Gorgens, Sydney Rosen, David Wilson, Yogan Pillay, Nicole Fraser‐Hurt

**Affiliations:** ^1^ Health Economics and Epidemiology Research Office Department of Internal Medicine School of Clinical Medicine Faculty of Health Sciences University of the Witwatersrand Johannesburg South Africa; ^2^ Department of Global Health Boston University School of Public Health Boston MA USA; ^3^ Department of Epidemiology Boston University School of Public Health Boston MA USA; ^4^ National Department of Health Pretoria South Africa; ^5^ The World Bank Group Washington DC USA

**Keywords:** adherence, ARV, counselling, differentiated care, South Africa, viral load

## Abstract

**Introduction:**

In response to suboptimal adherence and retention, South Africa’s National Department of Health developed and implemented National Adherence Guidelines for Chronic Diseases. We evaluated the effect of a package of adherence interventions beginning in January 2016 and report on the impact of Fast‐Track Treatment Initiation Counselling (FTIC) on ART initiation, adherence and retention.

**Methods:**

We conducted a cluster‐randomized mixed‐methods evaluation in 4 provinces at 12 intervention sites which implemented FTIC and 12 control facilities providing standard of care. Follow‐up was by passive surveillance using clinical records. We included data on subjects eligible for FTIC between 08 Jan 2016 and 07 December 2016. We adjusted for pre‐intervention differences using difference‐in‐differences (DiD) analyses controlling for site‐level clustering.

**Results:**

We enrolled 362 intervention and 368 control arm patients. Thirty‐day ART initiation was 83% in the intervention and 82% in the control arm (RD 0.5%; 95% CI: −5.0% to 6.0%). After adjusting for baseline ART initiation differences and covariates using DiD we found a 6% increase in ART initiation associated with FTIC (RD 6.3%; 95% CI: −0.6% to 13.3%). We found a small decrease in viral suppression within 18 months (RD −2.8%; 95% CI: −9.8% to 4.2%) with no difference after adjustment (RD: −1.9%; 95% CI: −9.1% to 5.4%) or when considering only those with a viral load recorded (84% intervention vs. 86% control). We found reduced crude 6‐month retention in intervention sites (RD −7.2%; 95% CI: −14.0% to −0.4%). However, differences attenuated by 12 months (RD: −3.6%; 95% CI: −11.1% to 3.9%). Qualitative data showed FTIC counselling was perceived as beneficial by patients and providers.

**Conclusions:**

We saw a short‐term ART‐initiation benefit to FTIC (particularly in districts where initiation prior to intervention was lower), with no reductions but also no improvement in longer‐term retention and viral suppression. This may be due to lack of fidelity to implementation and delivery of those components that support retention and adherence. FTIC must continue to be implemented alongside other interventions to achieve the 90‐90‐90 cascade and fidelity to post‐initiation counselling sessions must be monitored to determine impact on longer‐term outcomes. Understanding the cost‐benefit and role of FTIC may then be warranted.

## 1 INTRODUCTION

Extensive data from sub‐Saharan Africa since large‐scale antiretroviral therapy (ART) rollout began shows suboptimal retention in HIV care 1, 2, 3, 4, 5, 6. A particular point of concern is the period between treatment eligibility (previously based on CD4 cell count thresholds 7, 8) and initiation when limited loss is expected as patients are already enrolled in care and few barriers should remain to initiation. Still, up to one‐third 1, 9, 10 of patients are lost between these two points. Improving timely treatment uptake is thus a promising opportunity for intervention, offering the possibility of reducing the proportion of patients who start treatment with advanced disease, and the time patients have a detectable viral load and are infectious.

One reason patients leave care before ART initiation is the complicated steps required to initiate, including numerous counselling visits and laboratory tests. To mitigate the impact such delays have on retention, several studies have demonstrated that rearranging initiation algorithms to allow faster or same‐day initiation 11, 12, 13, 14, 15, 16, 17 improves ART uptake and outcomes 18. Most of these studies were done under trial conditions or in a few selected sites, however, leaving questions about generalizability to routine care.

South Africa has the largest HIV treatment programme worldwide 19, but elevated attrition rates 20, 21 threaten to reduce treatment benefits. To address concerns about retention and treatment adherence, in 2014 South Africa’s National Department of Health (NDOH) issued National Adherence Guidelines for Chronic Diseases 22. It calls for a minimum package of eight interventions targeting three groups of adult HIV patients, those who have: (1) newly tested and are initiating treatment (new patients); (2) initiated treatment and achieved viral suppression (stable patients); and (3) initiated treatment and have elevated viral loads or missed their schedule for clinical care (non‐stable patients) 23. NDOH began piloting these at “early learning sites” in 2015. One component is “Fast‐Track Treatment Initiation Counselling” (FTIC), developed and piloted by Médecins sans Frontiers (MSF) 17, designed to improve ART uptake and adherence by providing better adherence counselling without delaying treatment initiation. Prior to this evaluation facilities were fast‐tracking certain groups of patients (e.g. pregnant women, patients with CD4 <200 cells/mm^3^) for rapid ART initiation, as per the 2015 ART treatment guidelines. In September 2016, universal test‐and‐treat was introduced enabling HIV‐positive patients to initiate treatment on the day eligibility was ascertained. However, the content of counselling sessions has not been formally adapted to fit with these changes, nor have patient records or systems been fully updated to record these, and as such the extent and content of counselling provided remains unknown and likely varies by facility and healthcare provider.

We sought to determine if these interventions were effective at maintaining or increasing initiation rates, and determining whether adherence and retention post‐initiation is equivalent or better than under current guidelines. We worked with NDOH to conduct a cluster‐randomized mixed‐methods evaluation at early learning sites to inform future iterations of the adherence guidelines. Here we report the impact of FTIC on treatment initiation, viral suppression and retention.

## 2 METHODS

### 2.1 Study sites

We evaluated five of the adherence interventions (see protocol 23), including FTIC, in four provinces prioritized by NDOH (Gauteng, KwaZulu‐Natal, Limpopo, and North West). We selected three pairs of high‐volume HIV treatment facilities (>1000 ART patients) from a single district in each province (24 total) 23. Pairs of sites were matched on viral suppression, setting (rural/urban/formal/informal) and location (geographic proximity). Matching was done using clinic reported information just prior to the time of randomization. Within each pair, NDOH randomly allocated (using a computer programme) one to receive the adherence guidelines interventions and one to serve as a control (standard of care), giving us a cluster‐randomized evaluation design.

### 2.2 Study population

**Figure 1:**
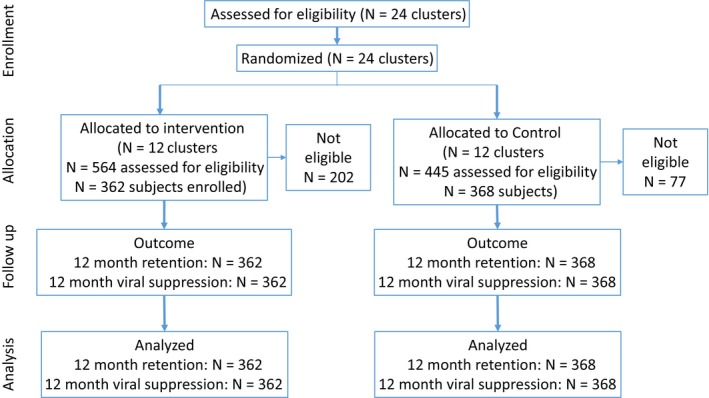
Flow diagram for Fast Track Initiation Counselling cohort creation and analyses.

We included patients eligible for FTIC under the National Adherence Guidelines, that is, those newly eligible to start ART under prevailing national guidelines (CD4 count <500, WHO Stage III/IV or clinician‐indicated eligibility, or after September 2016, from HIV diagnosis HIV positive once deemed eligible to start ART). We excluded patients <18 years old, pregnant women, and patients with TB or cryptococcal meningitis. Intervention participants were a sample of those eligible and recorded as having received the intervention (either in their patient file or on an FTIC register) at an intervention site. Controls were patients at control sites who would have been eligible for the intervention had it been offered, using the criteria above (Figure 1).

### 2.3 Standard‐of‐care

Prior to Adherence Guidelines introduction, standard care required four to six patient visits before ARVs were dispensed 11, 24. Visits typically included three individual and/or group counselling sessions and visits to receive laboratory results. While it was possible to initiate priority patients (e.g. very low CD4 counts or pregnant women) within one week, initiation typically took two to four weeks. With the introduction of test‐and‐treat it is likely that standard‐of‐care and timing of visits and counselling sessions changed to accommodate same day initiation, although to date no guidelines have been produced that document this. Recording of the timing and content of counselling sessions was poor at study sites, making it difficult to determine if or how the delivery of counselling sessions and standard‐of‐care changed at control sites.

### 2.4 Intervention

FTIC aims “to provide standardized education, counselling and support to patients on adherence without delaying treatment initiation and to assist the patient to develop their own adherence plan” 25. One counselling session is provided prior to ART initiation and one at initiation, with follow‐up sessions at one and two months post‐initiation. The aim was to allow initiation after two visits and within one week of ART eligibility while creating individualized patient adherence plans and ensuring post‐initiation adherence support 26. The study team played no role in intervention implementation and selection of patients who received FTIC was the responsibility of facility staff.

### 2.5 Enrolment and follow‐up

Enrolment was by record review. The study team had no contact with participants before short‐term endpoints were reached. At intervention and control sites lists of potentially eligible patients were identified from TIER.Net, South Africa’s HIV electronic medical record system. At intervention sites, lists were compared against registers or patient files to confirm patients received FTIC (determined by presence of their name on the FTIC counselling register and assuming all patients on the register received at least one session). At control sites lists were used to identify patient files to confirm eligibility. Patients were selected until the sample size was reached. Follow‐up data came from routinely collected clinic records including TIER.Net and patient files beginning at ART eligibility, with all patients followed for up to 18 months to assess primary outcomes. Unfortunately, recording of counselling sessions was poor on both the registers and patient files. Despite attempts to match registers to patient files it was difficult to determine which counselling sessions patients received.

### 2.6 Outcomes

Our short‐term primary outcome was the proportion of patients initiating ART within 30 days of eligibility. Our long‐term primary outcomes were the proportion of patients virally suppressed (any viral load <400 copies/mL^3^) within nine months of ART eligibility (defined here as our six month measure which allowed results within two to nine months). We excluded viral loads within two months of ART eligibility to prevent inclusion of baseline viral loads. Our secondary outcome was the proportion of patients initiating ART within one week of eligibility. As we had additional follow‐up data, however, we also present outcomes up to 18 months for viral suppression and at 12 months for retention. Suppression within 18 months was defined as any viral load <400 copies/mL^3^ within two to eighteen months of eligibility. If there were discordant results (one suppressed and one unsuppressed) the outcome was classified as suppressed. In order to understand whether this definition impacted the outcome measure, we compared the risk difference from this definition to the risk difference had we classified discordant viral load results based upon the suppression status of the last viral load measure in the data set. Twelve‐month retention was defined as being listed as retained in TIER.Net, defined as not transferred, become lost‐to‐follow‐up (failure to attend the clinic within 90 days of a scheduled ART visit), or died at 12 months. A patient could be retained without initiating ART. We considered those without a viral load as not suppressed and those who transferred as not retained.

### 2.7 Statistical methods

Existing clinic data 11, 27, 28, 29 suggested 30‐day ART initiation rates were roughly 60%. We hypothesized an absolute increase of 15% would be meaningful. With 24 clusters, an alpha = 0.05, power = 80%, and a coefficient of variation = 0.1, we required 300 patients/arm (600 total). We increased our sample size to up to 720 to allow for attrition.

For our outcomes we calculated crude risk differences (RD) and 95% confidence intervals (CI)accounting for site‐level clustering using linear regression and generalized estimating equations with an unstructured correlation matrix. Models were adjusted for age (18 to 29, 30 to 49, 50+), sex, baseline CD4 count (<200, 200 to 349, 350+) and WHO stage (I/II vs. III/IV).

As the sample size was moderate and our matching was coarse, baseline imbalances between arms could occur by chance. We therefore used a difference‐in‐differences (DiD) in addition to our original proposed analyses 30. We compared differences in outcomes between arms during the intervention period adjusted for differences prior to the intervention period by including data on patients at intervention and control sites from 1 January 2015 through 31 December 2015 (the “pre‐period”) who met the inclusion criteria. Data came from site electronic databases (TIER.Net), and we could therefore include the entire eligible pre‐intervention population. We fit a site‐level cluster‐adjusted linear regression:

$${\text{outcome}}_{\mathrm{ij}}=\beta_{1}+\beta_{1}*\text{period}+\beta_{2}*\text{FTIC}+\beta_{3}*\text{period}*\text{FTIC}+\theta *X_{\mathrm{ij}}+\mu_{\mathrm{ij}}$$

here outcome*_ij_* signifies a binary outcome indicator for the *i*th person in period *j*, period is an indicator variable (1 = intervention, 0 = pre‐intervention) and FTIC is an indicator variable for treatment arm (0 = control, 1 = intervention). *β*_3_ is the coefficient for the interaction between period and FTIC and represents the effect of FTIC adjusted for baseline outcome differences. *X*_ij_ indicates a vector of covariates for adjustment and *µ*_ij_ is the error term.

### 2.8 Qualitative data collection and analysis

At one pair of sites in each province we collected data from patients and providers to understand the implementation process and acceptability of interventions. Data were collected through quantitative surveys (n = 113), focus group discussions (FGDs) with patients (n = 22) and in‐depth interviews with healthcare providers (n = 48). Eligible patients were identified with assistance from facility staff and approached at clinic visits. Provider participants were purposively selected from each cadre of providers implementing adherence interventions. The estimated sample size was 70 patients at each of eight survey sites; ten patients per FGD (one FGD per site) focused on newly diagnosed patients; and interviews with up to six providers per site. Data were analysed in NVivo11© (Doncaster, Australia). Coding themes were identified *a priori* according to the evaluation questions with additional themes added as they emerged. Full details around the methods and results from this qualitative component are reported elsewhere 31, 32.

### 2.9 Ethical considerations

The trial was approved by the Human Research Ethics Committee of the University of the Witwatersrand Johannesburg and the Boston University Institutional Review Board. Both approved use of routine data collection with no patient contact for the impact evaluation and approved interviewing of both patients and providers after informed consent for the implementation evaluation. The study is registered at clincialtrials.gov (NCT02536768).

## 3 RESULTS

We included 730 patients eligible for FTIC between 8 January and 7 December 2016 (362 intervention, 368 control). There were 202 patients excluded from the intervention arm because they were not eligible (44% had not received the intervention, 30% did not meet other FTIC criteria and the remainder did not meet study eligibility criteria) and 77 excluded from the control arm (69% did not meet FTIC criteria). Participants were mostly under 40 years old (65%) and 61% were female (Table 1). Median CD4 count at ART initiation was low (224 cells/mL^3^). Intervention and control arms were largely balanced on baseline characteristics.

**Table 1:** Baseline characteristics of the enrolled Fast‐Track Treatment Initiation Counselling (FTIC) cohort by intervention and control status

**Characteristic**	FTIC Intervention N = 362 n (%)	FTIC Control N = 368 n (%)		FTIC Total N = 730 n (%)
Age (n = 730)				
18 to 29	95 (26%)	87 (24%)		182 (25%)
30 to 39	148 (41%)	142 (39%)		290 (40%)
40 to 49	79 (22%)	83 (23%)		162 (22%)
50+	40 (11%)	56 (15%)		96 (13%)
Gender (n = 730)				
Female	213 (58%)	213 (58%)		426 (58%)
Male	149 (42%)	155 (42%)		304 (42%)
CD4 Count (at ART initiation) (n = 716) (median, IQR)	205 (106 to 350)	238 (125 to 360)		224 (117 to 358)
TB status (n = 729)				
Current TB diagnosis	0 (0%)	0 (0%)		0 (0%)
No current TB diagnosis	361 (100%)	368 (100%)		729 (100%)

### 3.1 ART initiation

Within 30 days of eligibility, 83% of intervention and 82% of control arm patients (RD 0.5%; 95% CI: −5.0% to 6.0%) initiated ART (Table 2). There was wide variation by site ranging from 63% to 93% among control clinics and 62% to 100% among intervention clinics. Within seven days, 43% (n = 153) of intervention arm and 44% (n = 163) of control arm patients initiated ART. Median days to initiation was similar between arms (intervention eight days; IQR 6 to 16; control nine days; IQR 4 to 18) (Figure S1).

**Table 2:** ART initiation within 30 days for those eligible for Fast‐Track Treatment Initiation Counselling in the enrolled cohort

Intervention				Control			
Facility	N	Initiated within 30 days	% Initiated	Facility	N	Initiated within 30 days	% Initiated
GP Site 1	28	21	75.0	GP Site 4	29	22	75.9
GP Site 2	29	22	75.9	GP Site 5	30	24	80.0
GP Site 3	28	28	100.0	GP Site 6	30	19	63.3
LP Site 1	30	25	83.3	LP Site 4	35	27	77.1
LP Site 2	29	25	86.2	LP Site 5	29	26	89.7
LP Site 3	26	16	61.5	LP Site 6	30	22	73.3
NW Site 1	32	27	84.4	NW Site 4	30	25	83.3
NW Site 2	31	28	90.3	NW Site 5	30	26	86.7
NW Site 3	30	26	86.7	NW Site 6	29	26	89.7
KZN Site 1	33	28	84.8	KZN Site 4	34	31	91.2
KZN Site 2	34	29	85.3	KZN Site 5	32	27	84.4
KZN Site 3	30	23	76.7	KZN Site 6	30	28	93.3
Total^a^	360	298	82.8	Total	368	303	82.3
Risk difference				0.5% (−5.1% to 6.0%)			
Total (pre‐period)	5293	4719	89.2	Total (pre‐period)	4956	4611	93.0
Risk difference (pre‐period)				−3.9% (−5.0 to −2.8%)			
Difference in differences				4.4% (0.03% to 8.8%)			
Difference in differences (covariate adjusted)				6.3% (3.0% to 10.0%)			
Difference in differences (covariate adjusted and cluster adjusted)				6.3% (−0.6% to 13.3%)			

GP, Gauteng Province; LP, Limpopo Province; NW, North West Province; KZN, KwaZulu Natal Province.

a 2 subjects (0.2% of total sample size) do not have outcomes as they were not found in the TIER.Net dataset and the files were not able to be located during follow‐up data collection. Note that this is a crude analysis, without adjustment for clustering or covariates as is done below for the final model. All analyses are crude within the report unless otherwise specified

analyses are adjusted for clustering by site using a generalized estimating equation with site level clustering and an unstructured correlation matrix.

**Figure 2:**
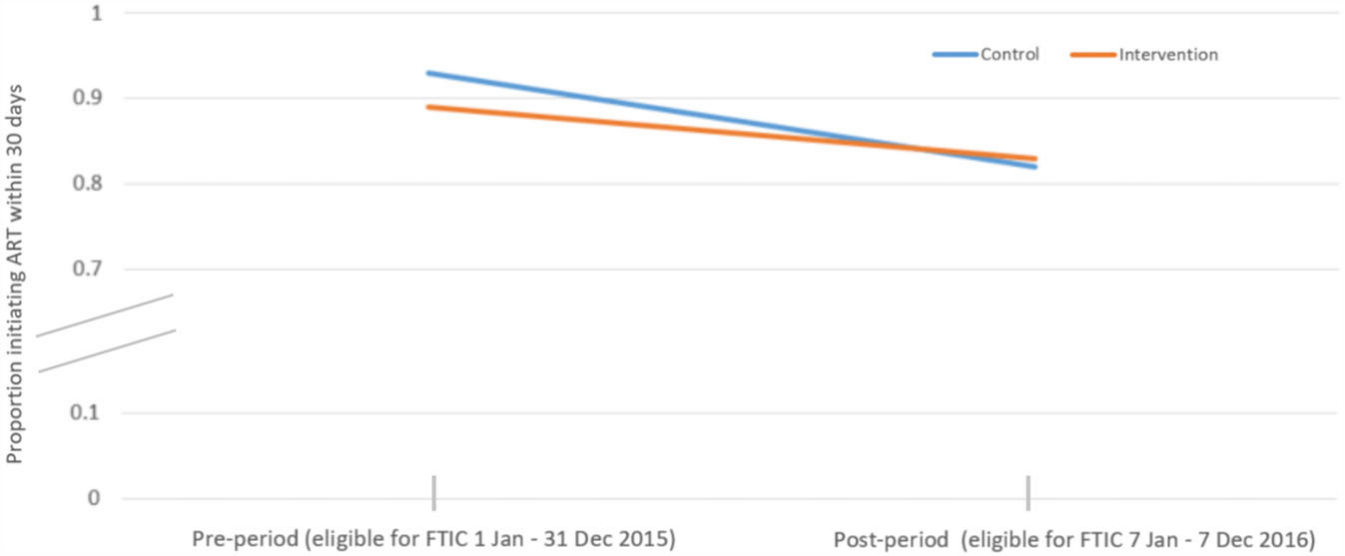
Difference‐in‐differences proportions in the pre‐ and post‐period for ART initiation within 30 days for those eligible for Fast‐Track Treatment Initiation Counselling cohort.

Although baseline characteristics were balanced, intervention sites had roughly 4% lower 30‐day ART initiation than control sites pre‐intervention (1 January 2015 to 31 December 2015) (RD −3.9%; 95% CI: −5.0 to −2.8%) (Table S1). Using difference‐in‐differences to adjust for this imbalance, intervention sites had 4.4% increased initiation compared to control sites (RD 4.4%; 95% CI: 0.03% to 8.8%) (Table 2, Figure 2). After adjusting for age, sex, baseline CD4 count, WHO stage and site‐level clustering, the difference increased slightly but confidence intervals widened (RD 6.3%; 95% CI: −0.6% to 13.3%)(full model in Table S2). FTIC was associated with a nearly 11 percentage‐point increase in initiation (RD 10.5%; 95% CI: −1.7% to 22.7%) in Gauteng, the province with the lowest pre‐intervention initiation rate (Table S3).

### 3.2 Viral suppression

Intervention sites had increased 6‐month viral suppression (RD: 7.0; 95% CI: −0.16% to 14.2%) versus control sites (Table S4). Viral suppression within 18 months was common among those with a viral load test (84% intervention vs. 86% control) but about 30% had no viral load recorded within 18 months. The median number of viral loads recorded during follow‐up was two (range 1 to 5) in both arms. In our enrolled cohort, we found a small crude decrease in 18‐month suppression comparing intervention to control arms but confidence intervals were wide (RD −2.8%; 95% CI: −9.8% to 4.2%; Table 3). The 18‐month suppression risk difference did not vary if the classification of discordant viral load results was based upon the last viral load measure (RD −2.8%; 95% CI: −10.0% to 4.3%). In the pre‐intervention period, we found no difference (RD −0.1%; 95% CI: −1.9% to 1.8%) between arms in 18‐month suppression (Table S5). Using difference‐in‐differences (Table 3) to adjust for baseline outcome imbalances, we again saw no real difference in suppression (RD: −2.7%; 95% CI: −10.1% to 4.6%). When further adjusting for site‐level clustering and individual baseline covariates this remained roughly null (RD: −1.9%; 95% CI: −9.1% to 5.4%) (Full model Table S6).

**Table 3:** Within 18‐month viral suppression (defined as any suppressed viral load between two and eighteen months) for those eligible for Fast‐Track Initiation Counselling in the Enrolled Cohort

Intervention					Control				
Facility	N	No VL	Suppressed	% Suppressed	Facility	N	No VL	Suppressed	% Suppressed
GP Site 1	28	9	14	50.0	GP Site 4	29	8	17	58.6
GP Site 2	30	5	18	60.0	GP Site 5	30	7	14	46.7
GP Site 3	28	11	13	46.4	GP Site 6	30	8	19	63.3
LP Site 1	30	7	22	73.3	LP Site 4	35	8	25	71.4
LP Site 2	29	7	21	72.4	LP Site 5	29	5	20	69.0
LP Site 3	26	15	10	38.5	LP Site 6	30	12	16	53.3
NW Site 1	32	9	20	62.5	NW Site 4	30	14	15	50.0
NW Site 2	31	5	22	71.0	NW Site 5	30	9	17	56.7
NW Site 3	30	11	14	46.7	NW Site 6	29	9	18	62.1
KZN Site 1	33	7	22	66.7	KZN Site 4	34	9	23	67.6
KZN Site 2	35	7	22	62.9	KZN Site 5	32	5	24	75.0
KZN Site 3	30	6	23	76.7	KZN Site 6	30	1	27	90.0
Total	362	99	221	61.0	Total	368	95	235	63.9
Risk difference^a^					−2.8% (−9.8% to 4.2%)				
Total (pre‐period)	5293	1637	3235	61.1	Total (pre‐period)	4956	1481	3032	61.2
Risk difference (pre‐period)					−0.1% (−1.9% to 1.8%)				
Difference in differences					−2.7% (−10.1% to 4.6%)				
Difference in differences (covariate adjusted)^b^					−2.7% (−9.7% to 4.2%)				
Difference in differences (covariate adjusted and cluster adjusted)^b^					−1.9% (−9.1% to 5.4%)				

GP, Gauteng Province; LP, Limpopo Province; NW, North West Province; KZN, KwaZulu Natal Province; VL, viral load.

a Note that this is a crude analysis, no adjustment for clustering or covariates as is done below for the final model

b Analyses are adjusted for clustering by site using a generalized estimating equation with site level clustering and an unstructured correlation matrix; note that sample size is smaller for the DiD covariate adjusted as those with missing data will drop out of the analysis.

### 3.3 Retention

At six months, retention was lower in the intervention compared to the control arm (RD: −7.2%; 95% CI: −14.0% to −0.4%) (Table S7). This was not sustained at 12 months, however, when retention was about 68% overall (Table 4). In a crude comparison, 12‐month retention was reduced in intervention sites (RD −2.5%; 95% CI: −9.3% to 4.5%). In the pre‐intervention period retention was somewhat higher than the intervention period (about 74%) and balanced across arms (control arm 73.0%, intervention arm 74.6%; RD: 1.6%; 95% CI: −0.1% to 3.3%) (Table S8). Using difference‐in‐differences, we saw a reduction in retention associated with FTIC (RD: −4.1%; 95% CI: −10.7% to −2.5%; Table 4) even when adjusting for clustering and baseline covariates (RD: −3.6%; 95% CI: −11.1% to 3.9%) (Full model Table S9).

**Table 4:** Retention (alive and in care) by 12 months for those eligible for Fast‐Track Initiation Counselling in the Enrolled Cohort

Intervention						Control					
Facility	N	Transfer	Died/LTF	Alive	% Retained	Facility	N	Transfer	Died/LTF	Alive	% Retained
GP Site 1	28	2	10	16	57.1	GP Site 4	28	0	10	18	64.3
GP Site 2	29	0	9	20	69.0	GP Site 5	30	2	6	22	73.3
GP Site 3	28	1	15	12	42.9	GP Site 6	30	0	7	23	76.7
LP Site 1	30	1	10	19	63.3	LP Site 4	35	1	7	27	77.1
LP Site 2	29	0	8	21	72.4	LP Site 5	29	3	2	24	82.8
LP Site 3	26	2	9	15	57.7	LP Site 6	30	0	9	21	70.0
NW Site 1	32	1	5	26	81.3	NW Site 4	30	5	14	11	36.7
NW Site 2	31	2	7	22	71.0	NW Site 5	30	3	11	16	53.3
NW Site 3	30	3	8	19	63.3	NW Site 6	29	3	7	19	65.5
KZN Site 1	33	3	7	23	69.7	KZN Site 4	34	1	8	25	73.5
KZN Site 2	34	3	9	22	64.7	KZN Site 5	32	1	8	23	71.9
KZN Site 3	30	2	3	25	83.3	KZN Site 6	30	3	2	25	83.3
Total^a^	360	20	100	240	66.7	Total^a^	367	22	91	254	69.2
Risk difference						−2.5% (−9.3% to 4.2%)					
Total (pre‐period)	5293	285	1060	3948	74.6	Total (pre‐period)	4956	422	915	3619	73.0
Risk difference (pre‐period)						1.6% (−0.1 to 3.3%)					
Difference in differences						−4.1% (−10.7% to 2.5%)					
Difference in differences (covariate adjusted)^b^						−4.1% (−11.4% to 3.2%)					
Difference in differences (covariate adjusted and cluster adjusted)^b^						−3.6% (−11.1% to 3.9%)					

GP, Gauteng Province; LP, Limpopo Province; NW, North West Province; KZN, KwaZulu Natal Province.

a Note that three individuals were not able to be linked to TIER.Net and were not found during file review so they do not have a retention outcome

b Analyses are adjusted for clustering by site using a generalized estimating equation with site level clustering and an unstructured correlation matrix; note that sample size is smaller for the DiD covariate adjusted as those with missing data will drop out of the analysis.

### 3.4 Survey and qualitative results

Overall 113 patients eligible for FTIC completed the cross‐sectional survey. Intervention arm patients (n = 55) reported a median of one fewer clinic visits prior to initiation (median 2 vs. 3) than control arm patients. More reported feeling involved in decisions affecting their care (51% vs. 41%). They were also more likely to receive counselling after ART initiation than control arm patients (27.3% vs. 13.8%). FGD patients receiving FTIC reported feeling supported by the additional counselling and indicated it helped with adherence. Providers noted that post‐initiation counselling was important to intervention success but also highlighted some confusion between FTIC and “treat all,” as these new guidelines were implemented at roughly the same time. Consequently providers were uncertain how the policies differed and unsure which patients should be prioritized for rapid initiation potentially explaining the drop in initiation seen during the post‐intervention period. To improve FTIC, patients and providers recommended initiating treatment at the first counselling session to further improve the initiation process.

## 4 DISCUSSION

Given the high risk of attrition from HIV care between ART eligibility and initiation 33, approaches to increase initiation are critical to achieving targets like 90‐90‐90. Achieving the “second 90” requires 90% of those who know their status initiate HIV treatment 34 and cannot be achieved if those eligible are lost before initiating. We evaluated Fast‐Track Initiation Counselling aimed at increasing ART uptake and reducing initiation times to one week or less. We found some short‐term but no long‐term benefit. Our finding suggests that for FTIC initiation benefits to translate into retention benefits, particularly in the test‐and‐treat era and initiation of patients not psychologically ready for treatment, FTIC support post‐initiation may need to be strengthened and paired with other effective interventions designed to support patients with adherence and retention.

We found that FTIC, when implemented as routine care without additional external resources, increased 30‐day initiation by approximately 6%. While the benefit was modest, it could be meaningful given initiation rates were high before the intervention – >80% in most control sites – making large differences unlikely. This is evidenced by the greater FTIC benefit in Gauteng (roughly 11 percentage‐points), where initiation without intervention was only about 70%. However, as this was not a pre‐specified comparison 35, 36, this improvement might be the result of early motivation and training at certain sites rather than a real change. The fact that ART initiation took an average of eight days, possibly indicates that even with expedited and reduced counselling prior to initiation, many patients still struggle or choose not to return in less than a week to initiate and may not feel ready to initiate the same day as diagnosis. Simply reducing the number of sessions prior to ART initiation to one before ART start, and adapting the counselling approach and content may also not be sufficient to lead to any large change, and the availability of counsellors might delay sessions and therefore treatment initiation.

Short‐term initiation increases did not lead to improved viral suppression or retention. We saw no difference in suppression between arms and possibly reduced 12‐month attrition though our findings were imprecise. There has been concern that if patients initiate treatment quickly (or early) attrition post initiation may increase 37, 38. Our results are not strong enough to confirm this, so continued monitoring of adherence to treatment schedules is important. While FTIC is designed to include post‐initiation counselling it is largely focused on initiation and showed success both here and in a previous study reported by Wilkinson et al. in Khayelitsha, South Africa 17. The lack of retention and suppression benefits points to a need to support newly initiating patients with additional interventions. Indeed, the RapIT study, showed improved short‐ and long‐term outcomes for patients offered same‐day initiation, but again higher post‐initiation attrition 11. Koenig et al. reported similar results from Haiti 12. A 2010 review showed that retention and suppression were increased with rapid initiation, though only by a small amount 39.

Our study adds to the evidence supporting rapid ART initiation 11, 12, 13, 14, 15, 16, 17. Unlike previous studies, though, ours was done under routine conditions, without external resources for implementation. The study conditions may thus explain why FTIC had only a very modest effect on uptake and also why the proportion of initiations apparently decreased when the intervention and test‐and‐treat were introduced. Recording of pre‐ART information on TIER.Net was a relatively recent addition prior to implementation of the adherence guidelines and as such we were aware that CD4 dates were often incorrectly captured making it seem as though patients were initiated the day of their CD4 test. Patients who did not initiate treatment could also be missed. Furthermore, like many cohorts 40, CD4 counts were low at ART initiation and some FTIC patients could have had a history of ART 41 and treatment default which would affect outcomes. This potentially suggests providers are continuing to prioritize the sickest patients, despite new guidelines.

Our evaluation suggested that FTIC benefits patients and providers by requiring fewer clinic visits before initiation. There was also a perceived increase in participation and decision‐making by patients who reported feeling better supported in their HIV care and being more satisfied with care. These benefits resonate with NDOH’s aims of efficiency gains in the ART programme, reducing unnecessary clinic visits and pre‐ART tracing, and delivery of more patient‐oriented care models. Even at intervention sites, feedback on the quality of care was not always positive and both patients and providers recommended improvements so that initiation could happen on the day of first counselling. Quality of follow‐up sessions was also seen as key to influencing long‐term adherence. Unfortunately, poor recording of counselling sessions meant we were unable to determine the quality of those sessions and survey outcomes suggest many patients may not have received sessions addressing issues of mobility and viral load. Fidelity to the intervention and recording delivery of sessions may be key to improving outcomes, otherwise FTIC differs little from pre‐ART counselling previously implemented. That said, the 6% ART initiation increase associated with FTIC comes on top of gains likely already made by accelerating treatment for the sickest patients. FTIC may increase clinic capacity to initiate the hundreds of thousands of new patients newly eligible for ART under “treat all,” many of whom will have relatively high CD4 counts and understanding the cost‐benefit of FTIC in light of this, is warranted.

Our study has numerous strengths, including a cluster‐randomized evaluation of an NDOH programmatic rollout and a difference‐in‐differences approach to account for baseline differences. It also has some important limitations. First, we used routinely collected data by design, as any attempt to collect data directly from patients could influence ART initiation. While we tried to improve routine data collection at study sites, we had some missing data and likely some misclassification. Second, because this was not an individually randomized trial, we were subject to potential baseline differences between arms. While our covariate and difference‐in‐differences adjustment attempted to mitigate this, some residual confounding may remain. Third, we did not control implementation of the intervention, which was conducted entirely by the NDOH and study clinics. We could therefore not identify which controls were likely to have been given the intervention beyond applying the eligibility criteria, nor were we able to control fidelity to implementing the intervention, determine why some eligible patients did not receive the intervention or how many counselling sessions intervention patients received. If the intervention was targeted towards those deemed more (or less) likely to succeed, some bias may have occurred. In addition, we defined retention as remaining in care at the original facility, but many patients move to new sites and are still retained 42, 43. Fourth, our sites were high volume HIV treatment facilities in high HIV‐burden districts and consequently the results may not generalize to smaller clinics in lower HIV‐burden districts. Finally, in the pre‐intervention period, some sites showed 100% percent ART initiation. This is unlikely and likely signals a failure to collect pre‐ART data, so patients only appeared in routine datasets on starting ART. However, as long as this was consistent between sites, it should not affect our study results. The prioritization of treatment initiation for different groups of patients prior to implementation of the Adherence Guidelines and the nationwide launch of the Universal Test and Treat strategy in September 2016 also potentially explains the confusion among some providers around prioritization of patients and how and when to complete counselling sessions and initiate ART.

## 5 CONCLUSIONS

We found that Fast‐Track Treatment Initiation Counselling was a viable approach to improving ART uptake with no reductions in, but also no evidence of a benefit in suppression or retention. As roll out of the Adherence Guidelines continues it will be important to strengthen post‐initiation counselling, to monitor fidelity to implementation, and to pair FTIC in newly initiated patients with interventions designed to improve retention and adherence so that any short‐term gains turn into long‐term benefits to both patients and clinics.

## Competing interests

The authors declare that they have no competing interests.

## Authors’ contributions

NF, SP, MP, MG, SR and MPF all contributed to developing the protocol. ANH, JM, DW and YP all contributed substantive changes to the protocol. MPF drafted the manuscript. All authors were involved in editing the final manuscript.

## Funding

This work was supported by World Bank Trust funds from several governments and Government of South Africa domestic health financing.

## Supporting information


**Figure S1.** Kaplan‐Meier curve showing days to ART initiation for all those patients eligible for Fast Track Treatment Initiation Counselling in the enrolled cohort.
**Table S1.** ART initiation within 30 days for all those who would have been eligible for Fast Track Treatment Initiation Counselling cohort in the period prior to the rollout of the interventions (1 January 2015 through 31 December 2015) (pre‐period)
**Table S2.** Regression coefficients for final model for difference‐in‐differences analysis of ART initiation within 30 days adjusted for site level clustering
**Table S3.** ART initiation within 30 days for those eligible for Fast Track Treatment Initiation Counselling cohort during the intervention period
**Table S4.** 6‐month viral suppression (defined as two to nine months) for those eligible for Fast Track Initiation Counselling in the enrolled cohort
**Table S5.** 18‐month viral suppression (defined as two to eighteen months) for all those who would have been eligible for Fast Track Initiation Counselling cohort in the period prior to the rollout of the interventions (1 January 2015 through 31 December 2015) (pre‐period)
**Table S6.** Regression coefficients for final model for difference‐in‐differences analysis of within 18‐month viral suppression (defined as two to eighteen months) adjusted for site level clustering
**Table S7.** Retention (alive and in care) at six months for those eligible for Fast Track Initiation Counselling in the enrolled cohort
**Table S8.** Long‐term retention outcome (alive and in care at 12 months) for all those who would have been eligible for Fast Track Initiation Counselling cohort in the period prior to the rollout of the interventions (1 January 2015 through 31 December 2015) (pre‐period)
**Table S9.** Regression coefficients for final model for difference‐in‐differences analysis of retention (alive and in care) at 12 months adjusted for site level clustering

## Data Availability

Data supporting the results in this paper are archived on The World Bank Documents and Reports repository and can be accessed at: https://microdata.worldbank.org/index.php/catalog/3520

## References

[1] Rosen S , Fox MP . Retention in HIV care between testing and treatment in sub‐Saharan Africa: a systematic review. PLoS Med. 2011;8:e1001056. 21811403 10.1371/journal.pmed.1001056 PMC3139665

[2] Mugglin C , Estill J , Wandeler G , et al. Loss to programme between HIV diagnosis and initiation of antiretroviral therapy in sub‐Saharan Africa: systematic review and meta‐analysis. Trop Med Int Heal. 2012;17:1509–20. 10.1111/j.1365-3156.2012.03089.x PMC3895621 22994151

[3] Fox MP , Rosen S . Retention of adult patients on antiretroviral therapy in low‐ and middle‐income countries: systematic review 2008. J Acquir Immune Defic Syndr. 2015;69:98–108. 25942461 10.1097/QAI.0000000000000553 PMC4422218

[4] Fox M , Rosen SB . Retention on antiretroviral therapy in low‐ and middle‐income countries: systematic review of papers and abstracts since 2008. Boston. 2014.

[5] Rosen S , Fox MP , Gill CJ . Patient retention in antiretroviral therapy programs in sub‐Saharan Africa: a systematic review. PLoS Med. 2007;4:e298. 17941716 10.1371/journal.pmed.0040298 PMC2020494

[6] Fox MP , Rosen S . Patient retention in antiretroviral therapy programs up to three years on treatment in sub‐Saharan Africa, 2007–2009: Systematic review. Trop Med Int Heal. 2010;15:1–15. 10.1111/j.1365-3156.2010.02508.x PMC2948795 20586956

[7] World Health Organization . Consolidated guidelines on the use of antiretroviral drugs for treating and preventing HIV infection: Recommendations for a public health approach. Geneva, Switzerland: World Health Organisation; 2013. 24716260

[8] World Health Organization . Antiretroviral therapy for HIV infection in adults and adolescents: Recommendations for a public health approach: 2010 revision. Geneva, Switzerland: World Health Organisation; 2010. 23741771

[9] Larson BA , Bistline K , Ndibongo B , et al. Rapid point‐of‐care CD4 testing at mobile HIV testing sites to increase linkage to care: an evaluation of a pilot program in South Africa. J Acquir Immune Defic Syndr. 2012;61:e13–7. 22659650 10.1097/QAI.0b013e31825eec60 PMC3458178

[10] Larson BA , Brennan A , McNamara L , et al. Lost opportunities to complete CD4+ lymphocyte testing among patients who tested positive for HIV in South Africa. Bull World Health Organ. 2010;88:675–80. 20865072 10.2471/BLT.09.068981 PMC2930360

[11] Rosen S , Maskew M , Fox MP , et al. Initiating antiretroviral therapy for HIV at a patient’s first clinic visit: the RapIT randomized controlled trial. PLoS Med. 2016;13:e1002015. 27163694 10.1371/journal.pmed.1002015 PMC4862681

[12] Koenig SP , Dorvil N , Dévieux JG , et al. Same‐day HIV testing with initiation of antiretroviral therapy versus standard care for persons living with HIV: a randomized unblinded trial. PLoS Med. 2017;14:e1002357. 28742880 10.1371/journal.pmed.1002357 PMC5526526

[13] Amanyire G , Semitala FCF , Namusobya J , et al.Streamlining antiretroviral therapy uptake: a stepped‐wedge cluster randomized trial. in: conference on retroviruses and opportunistic infections. Boston (Abstract 112), 2016.

[14] Geng EH , Havlir DV . The science of rapid start – from the when to the how of antiretroviral initiation. PLoS Med. 2017;14:e1002358. 28742819 10.1371/journal.pmed.1002358 PMC5526524

[15] Rosen S , Fox MP , Larson BA , et al. Simplified clinical algorithm for identifying patients eligible for immediate initiation of antiretroviral therapy for HIV (SLATE): protocol for a randomised evaluation. BMJ Open. 2017;7:e016340. 10.1136/bmjopen-2017-016340 PMC5726128 28554939

[16] Rosen S , Fox MP , Larson BA , et al. Accelerating the uptake and timing of antiretroviral therapy initiation in Sub‐Saharan Africa: an operations research agenda. PLoS Med. 2016;13:e1002106. 27505444 10.1371/journal.pmed.1002106 PMC4978457

[17] Wilkinson L , Duvivier H , Patten G , et al. Outcomes from the implementation of a counselling model supporting rapid antiretroviral treatment initiation in a primary healthcare clinic in Khayelitsha, South Africa. South African J HIV Med. 2015;16:1–7. 10.4102/sajhivmed.v16i1.367 PMC5843199 29568589

[18] World Health Organization (WHO) . Guidelines for Managing Advanced HIV Disease and Rapid Initiation of Antiretroviral Therapyy, July 2017. Geneva, 2017. 29341560

[19] Joint United Nations Programme on HIV/AIDS (UNAIDS) . The gap report – July 2014. Geneva, Switzerland, 2014.

[20] South Africa National Department of Health . Health Indicators Update: Antiretroviral Indicators. Pretoria, 2013.

[21] Fox M , Bor J , MacLeod W , et al. Is retention on ART underestimated due to patient transfers? Estimating system‐wide retention using a national labs database in South Africa. In: 21st International AIDS Conference. Durban, South Africa, 2016.

[22] National Department of Health . National adherence guidelines for chronic diseases (HIV, TB and NCDs), Version: 7 April 2015. Pretoria, 2015.

[23] Fox MP , Pascoe SJ , Huber AN , et al. Assessing the impact of the National Department of Health’s National Adherence Guidelines for Chronic Diseases in South Africa using routinely collected data: a cluster‐randomised evaluation. BMJ Open. 2018;8:e019680. 10.1136/bmjopen-2017-019680 PMC5781226 29358446

[24] Myer L , Zulliger R , Pienaar D . Diversity of patient preparation activities before initiation of antiretroviral therapy in Cape Town, South Africa. Trop Med Int Health. 2012;17:972–7. 22809271 10.1111/j.1365-3156.2012.03033.x

[25] National Department of Health Republic of South Africa . Standard Operating Procedures for Minimum Package of Interventions to Suport Linkage to Care, Adherence and Retention in Care, Adherence Guidelines for HIV, TB and NCDs. Pretoria, South Africa, 2016.

[26] Médecins Sans Frontières Khayelitsha . ART/TB/PMTCT initiation patient education and counselling model report and toolkit. Cape Town, 2015.

[27] Fox MP , Maskew M , MacPhail A , et al. Cohort profile: the Themba Lethu Clinical Cohort, Johannesburg, South Africa. Int J Epidemiol. 2013;42:430–9. 22434860 10.1093/ije/dys029 PMC3619949

[28] Fox MP , Shearer K , Maskew M , et al. HIV treatment outcomes after seven years in a large public‐sector HIV treatment program in Johannesburg, South Africa. AIDS. 2012;26:1823–8. 22739391 10.1097/QAD.0b013e328357058a PMC3600649

[29] Fox MP , Maskew M , Brennan AT , et al. Cohort profile: the Right to Care Clinical HIV Cohort, South Africa. BMJ Open. 2017;7:e015620. 10.1136/bmjopen-2016-015620 PMC5724130 28601835

[30] Dimick JB , Ryan AM . Methods for evaluating changes in health care policy: the difference‐in‐differences approach. JAMA. 2014;312:2401–2. 25490331 10.1001/jama.2014.16153

[31] The World Bank . Evaluation of the National Adherence Guidelines for Chronic Diseases in South Africa: Healthcare Provider Perspectives on Different Care Models, 2017. Washington, DC: World Bank; 2017.

[32] The World Bank . Evaluation of the national adherence guidelines for chronic diseases in South Africa: patient perspectives on differentiated care models, 2017. Washington, DC: World Bank; 2017.

[33] Kranzer K , Govindasamy D , Ford N , Johnston V , Lawn SD . Quantifying and addressing losses along the continuum of care for people living with HIV infection in sub‐Saharan Africa: a systematic review. J Int AIDS Soc. 2012;15:e17383. 10.7448/IAS.15.2.17383 PMC3503237 23199799

[34] UNAIDS . An ambitious treatment target to help end the AIDS epidemic. Geneva, Switzerland, Switzerland: 2014: p. 90–90‐90.

[35] Sun X , Briel M , Walter SD , Guyatt GH . Is a subgroup effect believable? Updating criteria to evaluate the credibility of subgroup analyses. BMJ. 2010;340:c117. 20354011 10.1136/bmj.c117

[36] Sun X , Briel M , Busse JW , et al. Credibility of claims of subgroup effects in randomised controlled trials: systematic review. BMJ. 2012;344:e1553. 22422832 10.1136/bmj.e1553

[37] Fox MP . Are we shifting attrition downstream in the HIV cascade? Lancet HIV. 2016;3(12):e554–5. 27771232 10.1016/S2352-3018(16)30149-7

[38] Fox MP , Rosen S . A new cascade of HIV care for the era of “treat all”. PLoS Med. 2017;14:e1002268. 28399160 10.1371/journal.pmed.1002268 PMC5388465

[39] Ford N , Kranzer K , Hilderbrand K , et al. Early initiation of antiretroviral therapy and associated reduction in mortality, morbidity and defaulting in a nurse‐managed, community cohort in Lesotho. AIDS. 2010;24:2645–50. 20980868 10.1097/QAD.0b013e32833ec5b2

[40] Carmona S , Bor J , Nattey C , et al. Persistent high burden of advanced HIV disease among patients seeking care in South Africa’s National HIV program: data from a Nationwide Laboratory Cohort. Clin Infect Dis. 2018;66 Suppl_2:S111. 29514238 10.1093/cid/ciy045 PMC5850436

[41] Osler M , Hilderbrand K , Goemaere E , et al. The continuing burden of advanced HIV disease over 10 years of increasing antiretroviral therapy coverage in South Africa. Clin Infect Dis. 2018;66:S118–25. 29514233 10.1093/cid/cix1140 PMC5850025

[42] Geng EH , Emenyonu N , Bwana MB , Glidden DV , Martin JN . Sampling‐based approach to determining outcomes of patients lost to follow‐up in antiretroviral therapy scale‐up programs in Africa. J Am Med Assoc. 2008;300:506–7. 10.1001/jama.300.5.506 PMC3711416 18677022

[43] Fox M , Bor J , Brennan AT , et al. How much is retention in HIV care underestimated due to patient transfers? Estimating retention using a national laboratory database in South Africa. PLoS Med. 2018;15:e1002589. 29889844 10.1371/journal.pmed.1002589 PMC5995345

