# Supplementary material for: Differentiated HIV care in South Africa: the effect of fast‐track treatment initiation counselling on ART initiation and viral suppression as partial results of an impact evaluation on the impact of a package of services to improve HIV treatment adherence
Source: J Int AIDS Soc. 2019 Nov 5;22(11):e25409. doi: 10.1002/jia2.25409 (PMC6831947; doi:10.1002/jia2.25409)
Supplement: Supplementary file 1 — Figure S1. Kaplan‐Meier curve showing days to ART initiation for all those patients eligible for Fast Track Treatment Initiation Counselling in the enrolled cohort. Table S1. ART initiation within 30 days for all those who would have been eligible for Fast Track Treatment Initiation Counselling cohort in the period prior to the rollout of the interventions (1 January 2015 through 31 December 2015) (pre‐period) Table S2. Regression coefficients for final model for difference‐in‐differences analysis of ART initiation within 30 days adjusted for site level clustering Table S3. ART initiation within 30 days for those eligible for Fast Track Treatment Initiation Counselling cohort during the intervention period Table S4. 6‐month viral suppression (defined as two to nine months) for those eligible for Fast Track Initiation Counselling in the enrolled cohort Table S5. 18‐month viral suppression (defined as two to eighteen months) for all those who would have been eligible for Fast Track Initiation Counselling cohort in the period prior to the rollout of the interventions (1 January 2015 through 31 December 2015) (pre‐period) Table S6. Regression coefficients for final model for difference‐in‐differences analysis of within 18‐month viral suppression (defined as two to eighteen months) adjusted for site level clustering Table S7. Retention (alive and in care) at six months for those eligible for Fast Track Initiation Counselling in the enrolled cohort Table S8. Long‐term retention outcome (alive and in care at 12 months) for all those who would have been eligible for Fast Track Initiation Counselling cohort in the period prior to the rollout of the interventions (1 January 2015 through 31 December 2015) (pre‐period) Table S9. Regression coefficients for final model for difference‐in‐differences analysis of retention (alive and in care) at 12 months adjusted for site level clustering [file JIA2-22-e25409-s001.docx]

**APPENDICES:**

**Figure S1: Kaplan-Meier curve showing days to ART initiation for all those patients eligible for Fast Track Treatment Initiation Counselling in the enrolled cohort**

[Insert Figure S1]

**Table S1. ART initiation within 30 days for all those who would have been eligible for Fast Track Treatment Initiation Counselling cohort in the period prior to the rollout of the interventions (Jan 1, 2015 through Dec 31, 2015)(pre-period)**

| **Intervention** | | | |  | **Control** | | | |
| --- | --- | --- | --- | --- | --- | --- | --- | --- |
| **Facility** | **N** | **Initiated within 30 days** | **% initiated** |  | **Facility** | **N** | **Initiated within 30 days** | **% initiated** |
| **GP Site 1** | 408 | 317 | 78% |  | **GP Site 4** | 534 | 472 | 88% |
| **GP Site 2** | 455 | 385 | 85% |  | **GP Site 5** | 653 | 575 | 88% |
| **GP Site 3** | 491 | 437 | 89% |  | **GP Site 6** | 397 | 355 | 89% |
| **LP Site 1** | 252 | 235 | 93% |  | **LP Site 4** | 199 | 145 | 73% |
| **LP Site 2** | 402 | 391 | 97% |  | **LP Site 5** | 195 | 190 | 97% |
| **LP Site 3** | 471 | 328 | 70% |  | **LP Site 6** | 203 | 197 | 97% |
| **NW Site 1** | 426 | 417 | 98% |  | **NW Site 4** | 363 | 362 | 100% |
| **NW Site 2** | 392 | 347 | 89% |  | **NW Site 5** | 256 | 227 | 89% |
| **NW Site 3** | 569 | 568 | 100% |  | **NW Site 6** | 670 | 668 | 100% |
| **KZN Site 1** | 389 | 310 | 80% |  | **KZN Site 4** | 246 | 243 | 99% |
| **KZN Site 2** | 877 | 827 | 94% |  | **KZN Site 5** | 1055 | 1007 | 95% |
| **KZN Site 3** | 161 | 157 | 98% |  | **KZN Site 6** | 185 | 170 | 92% |
| **Total** | **5293** | **4719** | **89%** |  |  | **4956** | **4611** | **93%** |
| **Risk difference** | -3.9% (-5.0 to -2.8%) | | | | | | | |

GP=Gauteng Province; LP=Limpopo Province; NW=North West Province; KZN=KwaZulu Natal Province

**Table S2- Regression coefficients for final model for difference-in-differences analysis of ART initiation within 30 days adjusted for site level clustering***

| **Generalized Estimating Equation Parameter Estimates** | | | | | | |  |
| --- | --- | --- | --- | --- | --- | --- | --- |
| **Parameter** |  | **Beta** | **Standard Error** | **95% Confidence Limits** | |  | |
| **Intercept (% initiation in the control group in the pre-period)** |  | 0.957 | 0.0169 | 0.9239 | 0.9902 |  | |
| **Intervention (vs control in the pre-period)** |  | -0.0268 | 0.0193 | -0.0647 | 0.011 |  | |
| **Post- vs pre-period (among the controls)** |  | -0.1209 | 0.0296 | -0.179 | -0.0629 |  | |
| **intervention*period (difference-in-differences estimate)** |  | 0.0634 | 0.0354 | -0.006 | 0.1327 |  | |
| **Female vs. Male** |  | -0.0013 | 0.0067 | -0.0143 | 0.0118 |  | |
| **Age 18-29.9 vs ≥ 50 years** |  | 0.0168 | 0.0127 | -0.0081 | 0.0416 |  | |
| **Age 30-49.9 vs ≥ 50 years** |  | 0.0086 | 0.0096 | -0.0103 | 0.0275 |  | |
| **ART initiation WHO Stage I/II vs III/IV** |  | 0.0037 | 0.0088 | -0.0136 | 0.021 |  | |
| **ART initiation CD4 < 200 vs ≥ 350** |  | 0.0103 | 0.0079 | -0.0052 | 0.0258 |  | |
| **ART initiation CD4 200-349 vs ≥ 350** |  | 0.0067 | 0.0058 | -0.0046 | 0.0181 |  | |

* Note that the effective sample size (n=646) is decreased due to missing values for CD4 count and WHO Stage. Site level clustering adjusted for using a generalized estimating equation with an unstructured correlation matrix.

**Table S3 - ART initiation within 30 days for those eligible for Fast Track Treatment Initiation Counselling cohort during the intervention period**

| **Intervention** | | | |  | **Control** | | | |
| --- | --- | --- | --- | --- | --- | --- | --- | --- |
| **Facility** | **N** | **Initiated within 30 days** | **% initiated** |  | **N** | **Initiated within 30 days** | **% initiated** | **Risk difference** |
| **Gauteng** | 85 | 71 | 84% |  | 89 | 65 | 73% | 10.5% (-1.7% to 22.7%) |
| **Limpopo** | 85 | 66 | 78% |  | 94 | 75 | 80% | -2.1% (-14.1% to 9.9%) |
| **North West** | 93 | 81 | 87% |  | 89 | 77 | 87% | 0.6% (-9.3% to 10.4%) |
| **KwaZulu-Natal** | 97 | 80 | 82% |  | 96 | 86 | 90% | -7.1% (-17.0% to 2.6%) |

**Table S4. 6-month viral suppression (defined as 2-9 months) for those eligible for Fast Track Initiation Counselling in the enrolled cohort**

| **Intervention** |  |  |  |  |  | **Control** |  |  |  |  |
| --- | --- | --- | --- | --- | --- | --- | --- | --- | --- | --- |
| **Facility** | **N** | **No VL** | **Suppressed** | **% Suppressed** |  | **Facility** | **N** | **No VL** | **Suppressed** | **% Suppressed** |
| **GP Site 1** | 28 | 11 | 7 | 25% |  | **GP Site 4** | 29 | 13 | 7 | 24% |
| **GP Site 2** | 30 | 9 | 12 | 40% |  | **GP Site 5** | 30 | 13 | 8 | 27% |
| **GP Site 3** | 28 | 13 | 12 | 43% |  | **GP Site 6** | 30 | 16 | 9 | 30% |
| **LP Site 1** | 30 | 10 | 19 | 63% |  | **LP Site 4** | 35 | 31 | 4 | 11% |
| **LP Site 2** | 29 | 10 | 19 | 66% |  | **LP Site 5** | 29 | 15 | 11 | 38% |
| **LP Site 3** | 26 | 19 | 5 | 19% |  | **LP Site 6** | 30 | 25 | 3 | 10% |
| **NW Site 1** | 32 | 14 | 16 | 50% |  | **NW Site 4** | 30 | 18 | 11 | 37% |
| **NW Site 2** | 31 | 9 | 19 | 61% |  | **NW Site 5** | 30 | 15 | 14 | 47% |
| **NW Site 3** | 30 | 17 | 10 | 33% |  | **NW Site 6** | 29 | 11 | 15 | 52% |
| **KZN Site 1** | 33 | 8 | 21 | 64% |  | **KZN Site 4** | 34 | 12 | 19 | 56% |
| **KZN Site 2** | 35 | 15 | 14 | 40% |  | **KZN Site 5** | 32 | 7 | 22 | 69% |
| **KZN Site 3** | 30 | 12 | 17 | 57% |  | **KZN Site 6** | 30 | 3 | 25 | 83% |
| **Total** | 362 | 147 | 171 | 47% |  | **Total** | 368 | 179 | 148 | 40% |
| **Risk difference*** | 7.0% (-0.16% - 14.2%) | | |  |  |  |  |  |  |  |

* Note that this is a crude analysis, no adjustment for clustering or covariates as is done for the final model. VL = viral load

GP=Gauteng Province; LP=Limpopo Province; NW=North West Province; KZN=KwaZulu Natal Province

**Table S5. 18-month viral suppression (defined as 2-18 months) for all those who would have been eligible for Fast Track Initiation Counselling cohort in the period prior to the rollout of the interventions (Jan 1, 2015 through Dec 31, 2015) (pre-period)**

| **Intervention** |  |  |  |  |  | **Control** |  |  |  |  |
| --- | --- | --- | --- | --- | --- | --- | --- | --- | --- | --- |
| **Facility** | **N** | **No VL** | **Suppressed** | **% Suppressed** |  | **Facility** | **N** | **No VL** | **Suppressed** | **% Suppressed** |
| **GP Site 1** | 408 | 119 | 224 | 54.9 |  | **GP Site 4** | 534 | 227 | 233 | 43.6 |
| **GP Site 2** | 455 | 123 | 249 | 54.7 |  | **GP Site 5** | 653 | 185 | 352 | 53.9 |
| **GP Site 3** | 491 | 156 | 275 | 56.0 |  | **GP Site 6** | 397 | 123 | 215 | 54.2 |
| **LP Site 1** | 252 | 55 | 182 | 72.2 |  | **LP Site 4** | 199 | 68 | 112 | 56.3 |
| **LP Site 2** | 402 | 113 | 263 | 65.4 |  | **LP Site 5** | 195 | 39 | 139 | 71.3 |
| **LP Site 3** | 471 | 204 | 243 | 51.6 |  | **LP Site 6** | 203 | 82 | 104 | 51.2 |
| **NW Site 1** | 426 | 113 | 283 | 66.4 |  | **NW Site 4** | 363 | 137 | 207 | 57.0 |
| **NW Site 2** | 392 | 108 | 252 | 64.3 |  | **NW Site 5** | 256 | 72 | 165 | 64.5 |
| **NW Site 3** | 569 | 288 | 250 | 43.9 |  | **NW Site 6** | 670 | 219 | 409 | 61.0 |
| **KZN Site 1** | 389 | 85 | 285 | 73.3 |  | **KZN Site 4** | 246 | 30 | 208 | 84.6 |
| **KZN Site 2** | 877 | 247 | 597 | 68.1 |  | **KZN Site 5** | 1055 | 269 | 738 | 70.0 |
| **KZN Site 3** | 161 | 26 | 132 | 82.0 |  | **KZN Site 6** | 185 | 30 | 150 | 81.1 |
| **Total** | 5293 | 1637 | 3235 | 61.1 |  | **Total** | 4956 | 1481 | 3032 | 61.2 |
| **Risk difference*** | -0.1% (-1.9% to 1.8%) | | |  |  |  |  |  |  |  |

* Note that this is a crude analysis, no adjustment for clustering or covariates as is done below for the final model.

GP=Gauteng Province; LP=Limpopo Province; NW=North West Province; KZN=KwaZulu Natal Province

**Table S6 – Regression coefficients for final model for difference-in-differences analysis of within 18-month viral suppression (defined as 2-18 months) adjusted for site level clustering***

| **Generalized Estimating Equation Parameter Estimates** | | | | | | |  |
| --- | --- | --- | --- | --- | --- | --- | --- |
| **Parameter** |  | **Beta** | **Standard Error** | **95% Confidence Limits** | | **p-value** | |
| **Intercept (% initiation in the control group in the pre-period)** |  | 0.6956 | 0.0281 | 0.6406 | 0.7506 | <.0001 | |
| **Intervention (vs control in the pre-period)** |  | 0.0405 | 0.0325 | -0.0232 | 0.1042 | 0.2129 | |
| **Post- vs pre-period (among the controls)** |  | 0.0018 | 0.0405 | -0.0776 | 0.0811 | 0.9653 | |
| **intervention*period (difference-in-differences estimate)** |  | -0.0186 | 0.037 | -0.091 | 0.0539 | 0.6158 | |
| **Female vs. Male** |  | 0.0613 | 0.0093 | 0.0432 | 0.0794 | <.0001 | |
| **Age 18-29.9 vs ≥ 50 years** |  | -0.0911 | 0.0174 | -0.1252 | -0.0571 | <.0001 | |
| **Age 30-49.9 vs ≥ 50 years** |  | -0.0292 | 0.0122 | -0.0531 | -0.0053 | 0.0165 | |
| **ART initiation CD4 < 200 vs ≥ 350** |  | -0.1744 | 0.0106 | -0.1951 | -0.1537 | <.0001 | |
| **ART initiation CD4 200-349 vs ≥ 350** |  | -0.0656 | 0.0129 | -0.0908 | -0.0403 | <.0001 | |

* Note that the effective sample size (n=648) is decreased due to missing values for CD4 count and WHO Stage. Site level clustering adjusted for using a generalized estimating equation with an unstructured correlation matrix.

**Table S7. Retention (alive and in care) at 6 months for those eligible for Fast Track Initiation Counselling in the enrolled cohort**

| **Intervention** |  |  |  |  |  | **Control** |  |  |  |  |  |
| --- | --- | --- | --- | --- | --- | --- | --- | --- | --- | --- | --- |
| **Facility** | **N** | **Transfer** | **Died/LTF** | **Alive** | **% retained** | **Facility** | **N** | **Transfer** | **Died/LTF** | **Alive** | **% retained** |
| **GP Site 1** | 28 | 1 | 10 | 17 | 60.7 | **GP Site 4** | 28 | 0 | 9 | 19 | 67.9 |
| **GP Site 2** | 29 | 0 | 8 | 21 | 72.4 | **GP Site 5** | 30 | 1 | 5 | 24 | 80.0 |
| **GP Site 3** | 28 | 1 | 13 | 14 | 50.0 | **GP Site 6** | 30 | 0 | 6 | 24 | 80.0 |
| **LP Site 1** | 30 | 1 | 8 | 21 | 70.0 | **LP Site 4** | 35 | 1 | 7 | 27 | 77.1 |
| **LP Site 2** | 29 | 0 | 8 | 21 | 72.4 | **LP Site 5** | 29 | 1 | 2 | 26 | 89.7 |
| **LP Site 3** | 26 | 2 | 9 | 15 | 57.7 | **LP Site 6** | 30 | 0 | 8 | 22 | 73.3 |
| **NW Site 1** | 32 | 1 | 5 | 26 | 81.3 | **NW Site 4** | 30 | 5 | 12 | 13 | 43.3 |
| **NW Site 2** | 31 | 2 | 6 | 23 | 74.2 | **NW Site 5** | 30 | 3 | 5 | 22 | 73.3 |
| **NW Site 3** | 30 | 3 | 8 | 19 | 63.3 | **NW Site 6** | 29 | 3 | 5 | 21 | 72.4 |
| **KZN Site 1** | 33 | 3 | 6 | 24 | 72.7 | **KZN Site 4** | 34 | 1 | 5 | 28 | 82.4 |
| **KZN Site 2** | 34 | 3 | 8 | 23 | 67.6 | **KZN Site 5** | 32 | 1 | 8 | 23 | 71.9 |
| **KZN Site 3** | 30 | 2 | 3 | 25 | 83.3 | **KZN Site 6** | 30 | 2 | 1 | 27 | 90.0 |
| **Total** | 360 | 19 | 92 | 249 | 69.2 | **Total** | 367 | 18 | 73 | 276 | 75.2 |
| **Risk difference** | | | | | | -6.0 (-12.5% to -0.5%) | | | |  |  |
| **Risk difference (pre-period)** | | | | | | 1.4% (-0.2% to 3.1%) | | | |  |  |
| **Difference in differences** | | | | | | -7.6% (-14.0% to -1.3%) | | | |  |  |
| **Difference in differences (covariate adjusted)*** | | | | | | -7.6% (-14.3% to -1.0%) | | | |  |  |
| **Difference in differences (covariate adjusted and cluster adjusted)*** | | | | | | -7.2% (-14.0% to -0.4%) | | | |  |  |

Note that three individuals were not able to be linked to TIER.Net and were not found during file review so they do not have a retention outcome.
* Analyses are adjusted for clustering by site using a generalized estimating equation with site level clustering and an unstructured correlation matrix; note that sample size is smaller for the DiD covariate adjusted as those with missing data will drop out of the analysis.

GP=Gauteng Province; LP=Limpopo Province; NW=North West Province; KZN=KwaZulu Natal Province

**Table S8. Long-term retention outcome (alive and in care at 12 months) for all those who would have been eligible for Fast Track Initiation Counselling cohort in the period prior to the rollout of the interventions (Jan 1, 2015 through Dec 31, 2015) (pre-period)**

| **Intervention** |  |  |  |  |  | **Control** |  |  |  |  |  |
| --- | --- | --- | --- | --- | --- | --- | --- | --- | --- | --- | --- |
| **Facility** | **N** | **Transfer** | **Died/LTF** | **Alive** | **% retained** | **Facility** | **N** | **Transfer** | **Died/LTF** | **Alive** | **% retained** |
| **GP Site 1** | 408 | 20 | 92 | 296 | 72.5 | **GP Site 4** | 534 | 29 | 182 | 323 | 60.5 |
| **GP Site 2** | 455 | 41 | 82 | 332 | 73.0 | **GP Site 5** | 653 | 34 | 158 | 461 | 70.6 |
| **GP Site 3** | 491 | 30 | 113 | 348 | 70.9 | **GP Site 6** | 397 | 14 | 107 | 276 | 69.5 |
| **LP Site 1** | 252 | 10 | 41 | 201 | 79.8 | **LP Site 4** | 199 | 17 | 28 | 154 | 77.4 |
| **LP Site 2** | 402 | 13 | 82 | 307 | 76.4 | **LP Site 5** | 195 | 4 | 17 | 174 | 89.2 |
| **LP Site 3** | 471 | 24 | 146 | 301 | 63.9 | **LP Site 6** | 203 | 6 | 45 | 152 | 74.9 |
| **NW Site 1** | 426 | 27 | 42 | 357 | 83.8 | **NW Site 4** | 363 | 114 | 35 | 214 | 59.0 |
| **NW Site 2** | 392 | 34 | 75 | 283 | 72.2 | **NW Site 5** | 256 | 24 | 47 | 185 | 72.3 |
| **NW Site 3** | 569 | 22 | 119 | 428 | 75.2 | **NW Site 6** | 670 | 64 | 120 | 486 | 72.5 |
| **KZN Site 1** | 389 | 19 | 78 | 292 | 75.1 | **KZN Site 4** | 246 | 18 | 18 | 210 | 85.4 |
| **KZN Site 2** | 877 | 39 | 173 | 665 | 75.8 | **KZN Site 5** | 1055 | 87 | 138 | 830 | 78.7 |
| **KZN Site 3** | 161 | 6 | 17 | 138 | 85.7 | **KZN Site 6** | 185 | 11 | 20 | 154 | 83.2 |
| **Total** | 5293 | 285 | 1060 | 3948 | 74.6 | **Total** | 4956 | 422 | 915 | 3619 | 73.0 |
| **Risk difference** | 1.6% (-0.1 to 3.3%) | | |  |  |  |  |  |  |  |  |

GP=Gauteng Province; LP=Limpopo Province; NW=North West Province; KZN=KwaZulu Natal Province

**Table S9 – Regression coefficients for final model for difference-in-differences analysis of retention (alive and in care) at 12 months adjusted for site level clustering***

| **Generalized Estimating Equation Parameter Estimates** | | | | | | |  |
| --- | --- | --- | --- | --- | --- | --- | --- |
| **Parameter** |  | **Beta** | **Standard Error** | **95% Confidence Limits** | | **p-value** | |
| **Intercept (% initiation in the control group in the pre-period)** |  | 0.7875 | 0.0243 | 0.7399 | 0.835 | <.0001 | |
| **Intervention (vs control in the pre-period)** |  | -0.0384 | 0.0299 | -0.097 | 0.0202 | 0.1986 | |
| **Post- vs pre-period (among the controls)** |  | 0.0161 | 0.0263 | -0.0353 | 0.0676 | 0.5389 | |
| **intervention*period (difference-in-differences estimate)** |  | -0.0374 | 0.0376 | -0.1112 | 0.0363 | 0.3199 | |
| **Female vs. Male** |  | 0.0364 | 0.0088 | 0.0191 | 0.0537 | <.0001 | |
| **Age 18-29.9 vs ≥ 50 years** |  | -0.0885 | 0.019 | -0.1257 | -0.0513 | <.0001 | |
| **Age 30-49.9 vs ≥ 50 years** |  | -0.0213 | 0.0143 | -0.0492 | 0.0067 | 0.1358 | |
| **ART initiation CD4 < 200 vs ≥ 350** |  | -0.0955 | 0.0089 | -0.113 | -0.078 | <.0001 | |
| **ART initiation CD4 200-349 vs ≥ 350** |  | -0.0405 | 0.0102 | -0.0605 | -0.0206 | <.0001 | |

* Note that the effective sample size (n=645) is decreased due to missing values for CD4 count and WHO Stage. Site level clustering adjusted for using a generalized estimating equation with an unstructured correlation matrix.
